# Incorporating mechanical strain in organs-on-a-chip: Lung and skin

**DOI:** 10.1063/1.5024895

**Published:** 2018-05-21

**Authors:** Olivier T. Guenat, François Berthiaume

**Affiliations:** 1 ARTORG Center, Medical Faculty, University of Bern, Bern, 3008 Switzerland; 2 Pulmonary Medicine Department, University Hospital of Bern, Bern, 3008 Switzerland; 3 Thoracic Surgery Department, University Hospital of Bern, Bern, Switzerland; 4 Department of Biomedical Engineering, Rutgers University, Piscataway, New Jersey, 08854, USA

## Abstract

In the last decade, the advent of microfabrication and microfluidics and an increased interest in cellular mechanobiology have triggered the development of novel microfluidic-based platforms. They aim to incorporate the mechanical strain environment that acts upon tissues and *in-vivo* barriers of the human body. This article reviews those platforms, highlighting the different strains applied, and the actuation mechanisms and provides representative applications. A focus is placed on the skin and the lung barriers as examples, with a section that discusses the signaling pathways involved in the epithelium and the connective tissues.

## I. INTRODUCTION

The recent emergence of organs-on-a-chip opens new opportunities in cell biology by uniquely reproducing key aspects of the *in-vivo* cellular microenvironment. One of these parameters is mechanical force, which imparts strain on cells and tissues and is an integral part of the environment that modulates the cellular phenotype. There is an extensive body of literature that describes the mechanisms whereby physical forces are transduced into biochemical signals that lead to responses at a single cell level.^1–3^ These responses, in turn, affect the function of multicellular systems (tissues), which is critical in health and disease. Physiological forces provide cues that superpose with biochemical signals that significantly impact morphogenesis^4^ during organ development, tissue homeostasis,^5^ and wound healing.^6^ Disease processes of fibrosis and cancer metastasis are also intimately linked to abnormal tissue mechanical properties.^5,7,8^

Mechanotransduction mechanisms operate through many of the familiar pathways that are involved in more “traditional” signaling triggered by biochemical factors. Yet, there have been few studies that examine the combined effects of physical and biochemical factors to ask questions about potential synergistic or antagonistic interactions. There is a need to fill this gap, as recent data have emerged suggesting that modulation of the mechanical environment can be purposely used to improve the wound healing response,^9^ either by promoting faster wound closure or by decreasing fibrosis.^10^ More generally, better characterization of the influence of mechanical forces is central to improving our understanding of pathophysiology and pathogenesis.^11^

Recent developments in the design of microphysiological “organ-on-a-chip” systems that recapitulate actual tissues on a small scale provide new opportunities for probing interactions between mechanical and biochemical signals. In this review, we describe the latest approaches used to recreate the *in vivo* biomechanical environment, in particular, mechanical strain, and how they can then be used to explore fundamental biological events, such as the wound healing response, as well as optimize potential drug screening tools. In contrast to the review by Schmitt *et al.*^12^ that focuses on the applicability of stretched systems for drug discovery, the present review focuses on microfluidic platforms able to create mechanical strain in *in-vitro* barriers. For the sake of conciseness, we focus primarily on the lung and skin systems as illustrative examples. After reviewing the types of microfluidic platforms reported and the associated type of mechanical strain, the last paragraph describes how the mechanical strain affects biological events via mechanotransduction signaling pathways.

## II. SKIN AND LUNG: EXAMPLES OF *IN-VIVO* BARRIERS EXPOSED TO MECHANICAL STRAIN

**FIG. 1.**
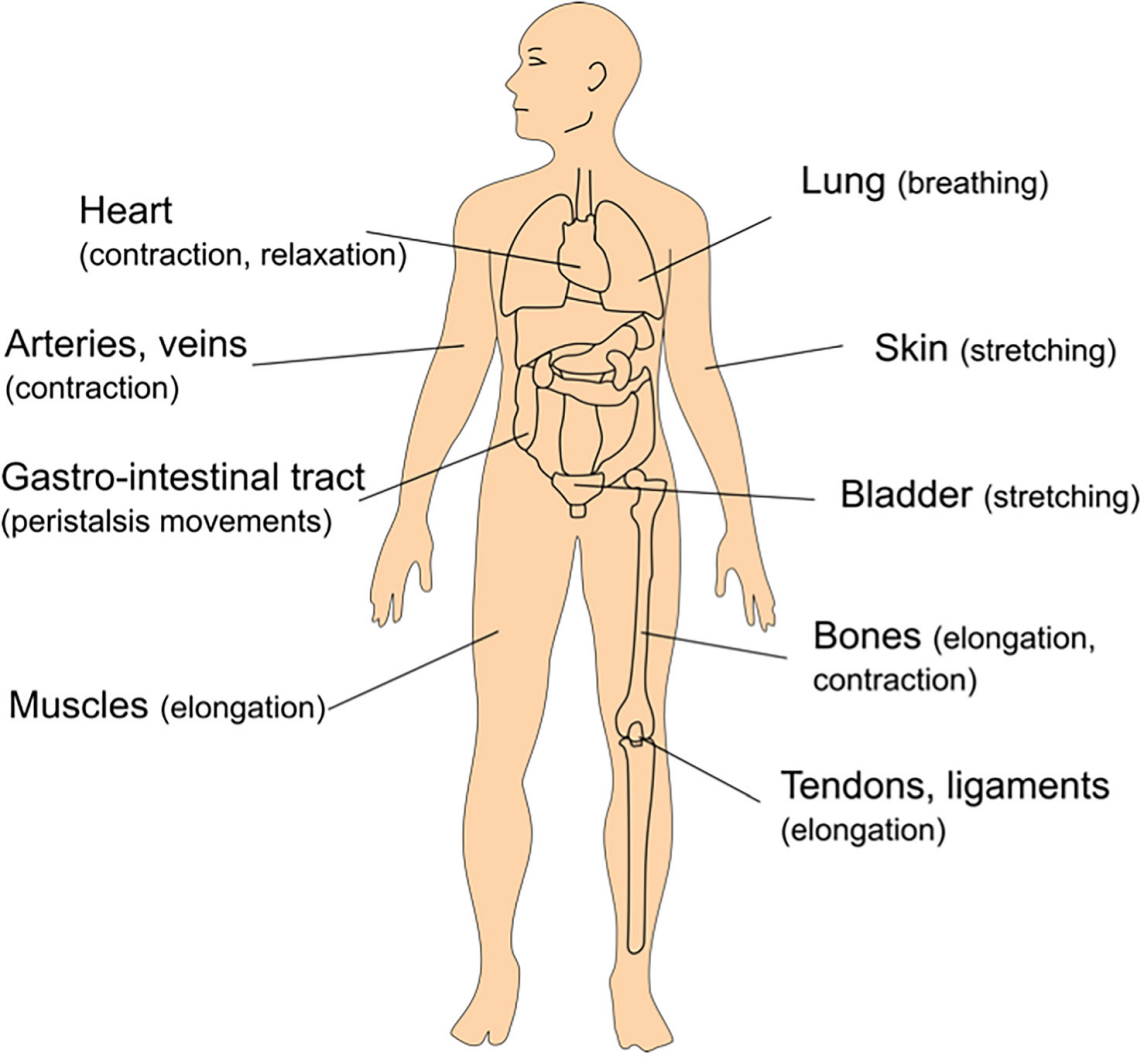
Examples of tissues exposed to mechanical strain in the human body.

A number of tissues and *in-vivo* barriers in the human body are exposed to mechanical strain. Mechanically active tissues, such as the heart or the muscles, are stretched continuously during daily activities. They in turn generate strain in other organs, such as the lungs, the skin, the tendons, the bones, and others. *In-vivo* barriers are particularly susceptible to such forces by their specific location at the interface with the outer world. As examples, the skin is exposed to stretching, the lung air-blood barrier to breathing movements, the gastro-intestinal tract to peristalsis motion, and the urothelium to stretching due to hydrostatic pressure. Figure 1 illustrates some examples of mechanical strain taking place in the human body.

The level of mechanical strain is very variable; it is a function of the tissue or barrier type and age, as well as other factors, such as the mechanical properties of the surrounding tissue [amount of extracellular matrix (ECM)]. It ranges between a few percent in the tendons and ligaments (2%–5%),^13^ and in the lung alveoli (4%–12%, see Sec. II B), and can reach up to tens of percent when muscles^14^ or skin is stretched (see Sec. II A). Pathological conditions can generate strain on tissues, for example, following hyperplasia,^15^ wound-healing, or tumor growth.

### A. Skin

Skin consists of three major layers: the epidermis, the dermis, and the hypodermis. The epidermis consists of multiple layers of epidermal cells (keratinocytes) forming tight junctions among themselves. At the dermal-epidermal junction, the first layer of keratinocytes functions as stem cells that self-renew and continuously generate new keratinocytes. The latter are pushed towards the skin surface and, in this process, undergo terminal differentiation into a cornified layer (the stratum corneum). This stratified organization underlies the main function of the epidermis, which is to form a barrier. Underneath is the dermis, which consists of a dense extracellular matrix network that harbors fibroblasts, blood vessels, and skin appendages (such as sweat glands, nerves, etc.). From a physical standpoint, the main function of the dermis is to provide mechanical integrity to the skin. In normal non-injured skin, the majority of compressive and tensile forces imparted onto the skin are borne by the extracellular matrix network in the skin dermis, with little force directly applied onto the resident fibroblasts or other structures.^16^ The hypodermis mainly consists of subcutaneous fat and serves as a shock absorber.

The mechanical stiffness of normal human dermis varies depending on the body location and depth inside the skin and has been reported to range between 0.1 and 10 kPa.^17^ Furthermore, the dermis is mechanically anisotropic due to preferential orientation of the collagen fibers along “Langer's lines,” topographical skin lines, which are generally parallel to the orientation of the underlying muscle fibers, and therefore the direction of stretch during joint movement. For example, Maiti *et al.* showed that forearm extension causes a tensile strain of 20 to 30% in the forearm skin^18^ but results in a relatively minimal level of stress. When skin is damaged deep enough to disrupt the underlying extracellular matrix, the wound fills with a fibrin clot with a stiffness between 0.01 and 1 kPa; because the force-bearing structure of the ECM is disrupted, part of the mechanical load is shifted onto the cellular network.^16^ Migrating fibroblasts form a network of differentiated myofibroblasts that promote wound contraction. These cells also secrete and restore the ECM. As this occurs, the load is shifted from the cellular network back to the ECM and hemostasis is restored. The resulting healed wound is however not identical to uninjured skin, as it consists of a scar which typically exhibits 10× more stiffness than normal skin.^19^

The local mechanical environment plays a critical role in whether healing results in a normal functioning scar or abnormal fibrotic scar (also known as hypertrophic scar and keloid). It is a well-known clinical observation that areas where skin is often under tension are more prone to develop a pathological process characterized by excessive fibrosis and wound contraction, which eventually limits joint movement.^20^ To minimize scarring, surgical incisions are usually performed along Langer's lines so as to minimally disturb the natural mechanical load distribution. Wounds that cut across those lines are subjected to greater mechanical tension and are more likely to result in excessive scarring.

Externally applied mechanical forces have been used as a therapeutic tool to improve the wound healing response. For example, recent studies have shown decreased hypertrophic scar formation when devices as simple as tape or silicone gel sheeting are applied over the wound area because they divert some of the mechanical load away from the fibroblast.^21,22^ Silicone balloon expanders have been used for decades to promote skin regeneration in cases where not enough skin is available before plastic and reconstructive procedures.^23^ In this case, the application of tensile force promotes the formation of new tissues. In cases of impaired wound healing (such as in diabetes), a popular method to enhance the healing response is the application of vacuum over the wound site (in a procedure called vacuum-assisted wound closure or negative pressure wound therapy). Although the mechanism of action is likely multifaceted, there are strong suggestions that mechanical deformation and stretching play an important role.^24^

The general consensus is that in skin, mechanical tension promotes tissue formation. As will be discussed later (see Sec. IV), epidermal keratinocytes and dermal fibroblasts can sense these mechanical effects, which, together with soluble and immobilized signal cues, are responsible for the observed wound healing response. Mechanically competent skin-on-a-chip platforms make it possible to investigate these interactions and provide a controlled environment to better understand the conditions that provide better wound healing responses.

### B. Lung

The human lung is an organ with a complex architecture that comprises two tree-like structures, the airway and the vascular trees. The airways divide into a succession of 23 generations of airways that terminate with tiny alveoli, where the gas exchange between air and blood takes place. The arterial pulmonary vasculature^25^ splits into smaller vessels that end in a large network of capillaries enrobing the alveoli. Oxygen diffuses through the ultra-thin alveolar barrier and is taken up by red blood cells, while carbon dioxide is simultaneously released. The alveolar epithelium consists of type I and type II lung alveolar epithelial cells. Type I epithelial cells are squamous and constitute 95% of the lung alveolar surface area. These ultra-thin and broad cells facilitate the rapid diffusion of oxygen and carbon dioxide^26^ from the air to the capillaries and vice-versa. In contrast, type II alveolar epithelial cells are small and cuboidal and only make up a small portion of the alveolar surface. They secrete a lipoprotein, called surfactant, that reduces the surface tension of the alveoli and prevents atelectasis, the collapse of the lung alveoli. The lung alveolar epithelial barrier results from tight junctions created by intercellular proteins, mostly from the claudins^27^ and occludens families.^28^ Epithelial cells are polarized with the apical side in contact with air and the basal side in contact with the ultra-thin basement membrane made of collagen and elastin. It is sandwiched between the epithelium and the blood capillaries. The endothelial barrier shows nearly identical intercellular junctions like the epithelial counterpart and controls the exchange of fluid and solutes with the surrounding tissue.^29^ Adjacent alveoli are separated by the alveolar septum that consists of the basement membrane and of the connective tissue. Immune cells, mostly macrophages and dendritic cells, are located in this environment in close proximity to the air-blood barrier. They communicate with epithelial cells to respond to infection, epithelial barrier damage, and pathogen clearance.^30^

In addition to these physiological aspects, this complex environment is continuously exposed to a number of physical forces that are transmitted across length scales, from the organ-to-the microscale level.^31^ These forces include the mechanical strain induced by breathing motions, the shear stress created by the blood, and interstitial flow and surface tension, in particular, in the alveoli.^32^

At rest, a healthy human lung typically inflates at a respiratory rate of 10–12 breaths per minute. The question on how the alveoli deforms—like a pre-stressed balloon or like an accordion-like structure^33^—and thus whether the volume fluctuation translates to a stretching of the epithelial cells is still a matter of debate. Roan and Waters suggest that the alveoli expansion results from a combination of mechanisms involving isotropic expansion and shape change.^34^ Using optical sectioning microscopy, Perlman and Bhattacharya observed non-uniform alveoli expansion in rat lungs, with type I alveolar epithelial cells being stretched more than type II cells.^35^ During normal breathing, the basement membrane is distended to about 4% linear strain on average. During a deep inspiration, the distal tissues undergo a larger deformation of up to 12% linear strain.^3^ The distension level of the lung alveoli strongly depends on the mechanical properties of the lower airways. The stiffness of a healthy human lung is about 2 kPa. In diseases, such as lung fibrosis, the tissue stiffens due to a pathological accumulation of ECM proteins secreted by epithelial cells and fibroblasts. This results in stiffer fibrotic tissues, typically with stiffness around 16 kPa.^36^ This leads to an increase in airway resistance to inflation^37^ and thus to a decrease in the mechanical strain in the parenchymal area. The lungs can also be exposed to larger pathophysiological strains (>20% linear strain) during positive mechanical ventilation.^38^ The lung of patients suffering from acute respiratory distress syndrome (ARDS) can be partly obstructed, which causes overdistension of the lung. This has been reported to augment the disruption of the lung alveolar epithelial barrier leading to infiltration and even to fibrosis.^39^

Our knowledge on the impact of the mechanical strain on lung cells has increased importantly in the last two decades.^3,12^ However, relatively little has been done to assess the effects of mechanical forces on the pathophysiology of parenchymal lung diseases.^40^ In lung fibrosis, mechanical forces are known to affect the development and the progression of the disease,^41^ but recent findings regarding the effects of mechanical cues^42,43^ suggest that much remains to be done to understand and find therapeutic options for this illness. The advent of organs-on-a-chip technologies that make it possible for the first time to mimic mechanical strain on *in-vitro* barriers may help elucidate mechanistic pathways involved in such diseases (see Sec. III B).

## III. MICROFLUIDIC PLATFORMS AIMED AT REPRODUCING THE *IN-VIVO* MECHANICAL STRAIN

*In-vitro* assays have been used early on to reproduce the mechanical strain that takes place in various tissues. Many research groups either produced their own stretching platform^44^ or used the Flexcell system (Flexcell International Corp.) that has been commercially available for over 30 years. With the advent of microfluidics and microfabricated systems and renewed interest in cellular mechanobiology, a number of microfluidic platforms have been reported to reproduce various types of mechanical strains. The introduction of poly-dimethylsiloxane (PDMS) soft lithography^45^ enabled the development of cost-efficient microsystems, with arrays of microwells using less cells and suitable for parallel experimentations. Furthermore, in addition to the mechanical strain, other aspects of the cellular environment were integrated in these platforms, such as shear stress (perfusion) or more recently *in-vitro* barriers with flexible and porous membranes.

In this section, we review two categories of microfluidic platforms aimed at mimicking the mechanical strain (Table I). The first involves devices equipped with microfabricated membranes, on which cells are cultured and mechanically stretched. The second group summarizes microfluidic devices that in addition to the mechanical strain integrate an ultra-thin, porous, and elastic membrane used as an *in-vitro* barrier. This sorting was chosen, as the potential of the platforms from the second category is much greater as discussed later (see Sec. III B). The type of mechanical strain is another important aspect that will be discussed in this section, as the diversity of the devices and of strain applied makes cross-comparison between studies difficult.

**TABLE I.** Microfluidic platforms aimed at reproducing mechanical strain *in-vitro*. The first category summarizes devices aimed at stretching cells cultured on a substrate, while the second category enables emulating *in-vivo* barriers, based on a thin, elastic, and porous membrane.

Device type	Type of strain			Cell culture support			Actuation	Applications	Year	References
	Direction	Level (in %)	Frequency	Membrane	Array	Coating				
Non barrier	Uni-axial	10% (linear strain)	Cyclic (1 Hz)	0.5–1 mm thick PDMS substrate	2	Gelatin	A precision linear motor applies cyclic stretch to the PDMS device.	Differentiation of murine embryonic stem cells in cardiomyocytes upon cyclic strain.	2011	Wan *et al.* (46)
		4% (linear strain)	Cyclic (0.5 Hz, 10 cycles)	Thin PDMS membrane with 10 *μ*m grooves	1	ProNectin F	Two linear actuators operated with a syringe pump.	Ca2+ signaling of tenocytes in response to cyclic strain.	2013	Wall *et al.*^62^
		3–7% (linear strain)	Cyclic (2 Hz)	100 *μ*m thick PDMS membrane	1	Fibronectin	Stretching by actuation of thin walls connected to adjacent channels with cyclic vacuum.	Provide mechanical, electrical, and biochemical stimulation to mesenchymal stem cells.	2015	Pavesi *et al.*^86^
	Bi-axial (xy)	0 up to 60% (surface strain)	Cyclic (1.3 Hz)	100 *μ*m to 330 *μ*m thick PDMS membrane	1	Fibronectin	Combination of hydrodynamic pressure & mechanical pressure with a post.	Hemodynamic stimulation of cardiomyocytes	2010	Giridharan *et al.*^55^
	Bi-axial (xz)	2–20% (circumferential strain)	Cyclic (1 Hz)	35 *μ*m thin PDMS membrane	5	Collagen, fibronectin, gelatin	Hydrodynamic actuation (microfluidic channel filled with liquid).	Mimic the circumferential strain to which small blood vessels are exposed	2012	Zhou *et al.*^47^
	Equi-bi-axial (xy)	2–15% (circumferential & radial strain)	Cyclic (1 Hz)	15 *μ*m thin PDMS membrane	9 × 12	Collagen	Positive pressure created by flat posts pushed against the culturing membrane.	Activation of the canonical Wnt/b-catenin signaling pathway in cardiac valve mesenchymal progenitor cells	2010	Moraes *et al.*^50^
		1, 2, 4, 6% (linear strain)	Cyclic (1 Hz)	150 *μ*m thick PDMS membrane	5 × 5	Fibronectin	The PDMS membrane is stretched with a vacuum around cylindrical, flat micropillars.	Strain of C2C12 skeletal myoblasts.	2012	Simmons *et al.*^53^
	Tri-axial	17–20% (surface strain)	Cyclic (0.2, 1, 5 Hz)	100 *μ*m thick PDMS membrane	3 × 8	Fibronectin	Mechanical movements of small pins that deflect the membrane (Braille display).	Strain of human dermal microvascular endothelial cells	2008	Kamotani *et al.*^48^
		15–50% (linear strain)	Cyclic (0.2–0.3 Hz)	100 *μ*m thick PDMS membrane	1	Fibronectin	Fluidic pressure created by a syringe pump (negative pressure).	Combined effects of fluid and solid mechanical stress on alveolar cells (mimic pathophysiology of ventilator induced lung injury).	2011	Douville *et al.*^54^
		3 and 12% (circumferential & radial strain)	Cyclic (1 Hz)	45 *μ*m and 100 *μ*m thin membranes made of PDMS with PU coating	12 × 9	Collagen or fibronectin	Pneumatic positive pressure (microfluidic channel filled with air).	Investigation of mechanobiological response profiles of valvular interstitial cells.	2013	Moraes *et al.*^49^
		6% (linear strain)	Cyclic (1 Hz)	10 *μ*m thin PDMS membrane	1	Collagen	Pneumatic negative pressure created below the thin membrane.	Study of cellular (MSC) responses to cyclical hypoxia and stretch.	2016	Campillo *et al.*^56^
		2.2–3.5% (linear strain)	Cyclic (0.33 Hz)	130 *μ*m thick PDMS membrane	5 × 6	None	Pneumatic negative pressure (vacuum created in microchannels).	Effect of mechanical strain on proliferation and differentiation of mesenchymal stem cells.	2014	Gao *et al.*^52^
		12–20% (circumferential & radial strain)	Cyclic (1 Hz)	35, 55, 75 *μ*m thin membrane in PDMS	32	Fibronectin	Pneumatic positive pressure to deflect the PDMS membrane.	Investigation on effect of cyclic stretch on membrane permeability of both healthy and dystrophic myotube.	2015	Michielin *et al.*^51^
*In-vitro* barrier	Uniaxial	5–15% (linear strain)	Cyclic (0.2 Hz)	10 *μ*m thin, PDMS membrane with 10 *μ*m wide pentagonal pores	1	Fibronectin or collagen	Stretching by actuation of thin walls connected to adjacent channels with cyclic vacuum [Fig. 2(a)].	Lung-on-a-Chip: Mimic the lung alveolar barrier and investigate the effects of the mechanical strain on toxic and inflammatory response.	2010	Huh *et al.*^58^
	Tri-axial	21% (surface strain)	Cyclic (0.2 Hz)	3 *μ*m thin, PDMS membrane with 3 or 8 *μ*m pores	3	Fibronectin	Stretching by indirect actuation using a bio-inspired microdiaphragm [see Fig. 2(b)].	Lung-on-a-Chip: Mimic the lung alveolar barrier and investigate the effects of the mechanical strain on primary lung alveolar cells.	2015	Stucki *et al.*^59^

### A. Stretching platforms without a porous membrane

A number of microfluidic-based platforms aimed at reproducing mechanical strain either in one, two, or three directions were reported in the last decade (Table I). The versatility of the technologies used to design and produce these systems makes it possible to easily create more complex structures, such as the coupling of a microfluidic channel with a stretchable membrane. An additional advantage of those systems is the ability to miniaturize the size of the culture well and to simultaneously create an array of such wells in order to increase the number of biological experiments. Arrays from a few parallel wells^46,47^ of up to tens of microwells^48–51^ were reported. The technologies used to produce these devices can be scaled up in view to integrate these systems in drug screening programs. As the number of wells increases, the stretching actuation needs to be kept as simple as possible to make the handling of such systems by non-experts as easy as possible. Takayama and colleagues were the first to report about a microfluidic stretching platform.^48^ They used an array of individually addressable pins to push upon a culturing elastic membrane to create a mechanical strain. Moraes *et al.* employed the same technique to generate a uniform equi-bi-dimensional strain using flat pillars.^50^ Pneumatic^49,51–53^ or hydrodynamic^47,54^ actuations are other popular techniques to create mechanical strain. Their simplicity allows filling a channel either with a gas or a liquid to inflate (positive pressure) or deflect (negative pressure) a thin membrane, on which cells are cultured. The culturing membranes were all based on PDMS, a material that among others exhibits great mechanical properties (its Young's modulus is typically around 1 MPa) and that can easily be deformed. To make the PDMS less oxygen permeable and reduce the absorption and adsorption of small molecules, PDMS was dip-coated with a thin layer of polyurethane to improve cell adhesion.^49^ Biological questions that have been investigated with these technologies cover a wide range: effect of the mechanical strain on the differentiation of embryonic stem cells; stimulation of cardiomyocytes^46^ or other cardiac cells;^55^ mimicking the rhythmic beating of small blood vessels;^47^ and emulating the breathing motions of the lung.^54,56^ Recently, based on the lung-on-a-chip system reported by Huh and colleagues,^58^ Kamm's group reported a system able to provide mechanical, biochemical, and electrical stimulation to both guide and monitor the morphological and genetic modifications of human bone marrow mesenchymal stem cells.^86^

### B. Stretching platforms with a porous membrane

In sharp contrast to the devices presented above, platforms equipped with a porous membrane make it possible to evaluate transport across the cultured cell layers, thus more closely mimicking *in-vivo* barriers. Cells can be cultured on both sides of the membrane, through which they can touch each other and/or communicate via paracrine and endocrine signaling. Furthermore, those systems enable reproduction of the air-liquid interface by providing nutrients to the cells cultured on the apical side via the porous membrane. This is a key feature that is needed to promote epithelial differentiation in skin and lung systems. Furthermore, the cells on one or both sides of the porous membrane can be exposed to shear stress by perfusing cell culture medium, blood, or other biological solutions.

**FIG. 2.**
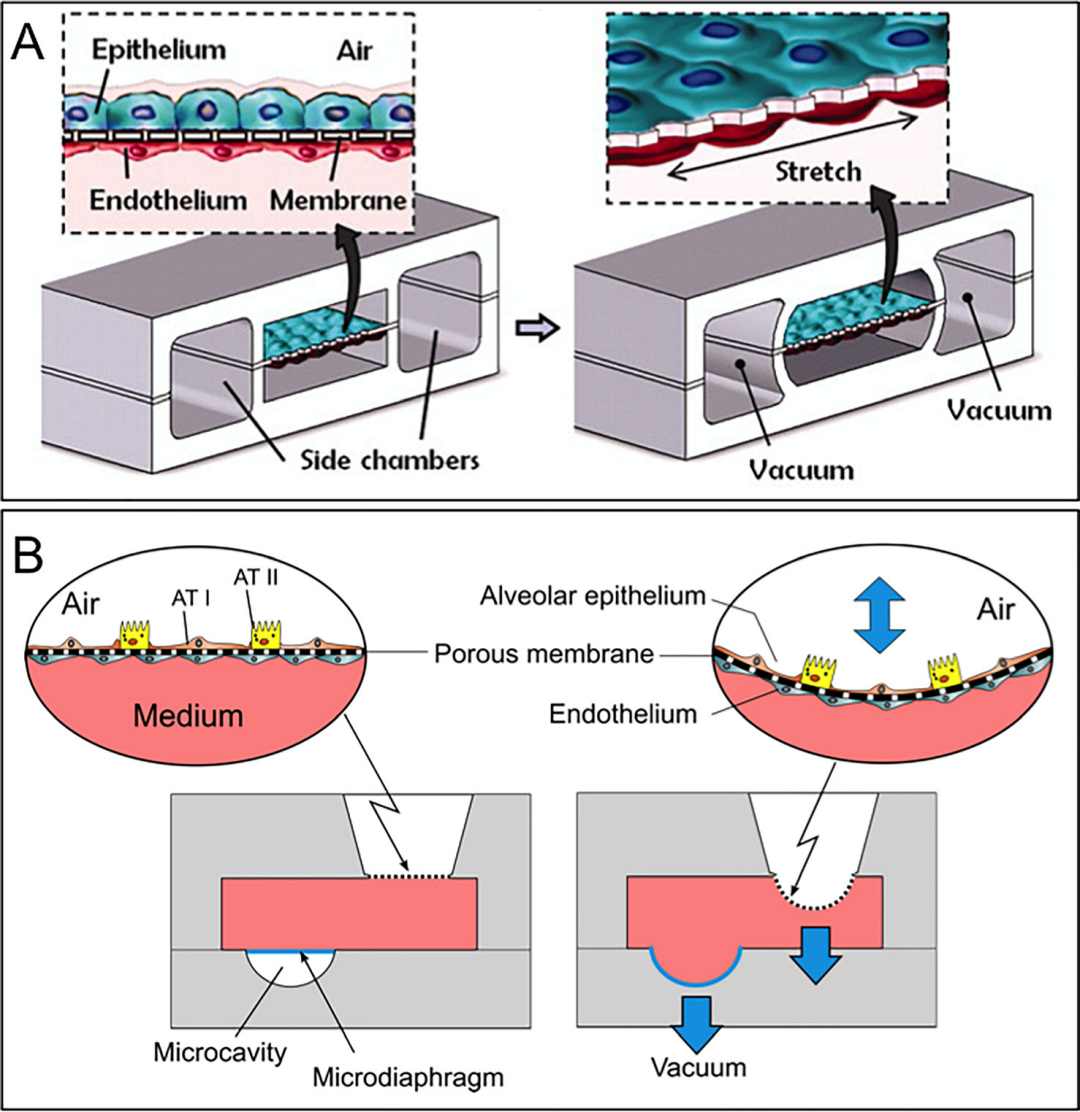
Lung-on-chips with a thin, elastic, and porous membrane used to culture cells on both sides of the membrane. Type I (ATI) and type II (ATII) lung alveolar epithelial cells are cultured on the apical side of the membrane, whereas endothelial cells are cultured on its basolateral side. (a) 10 *μ*m thin, membrane stretched with a uniaxial strain. Reproduced with permission from Huh *et al.*, Science **328**(5986), 1662–1668 (2010). Copyright 2010 American Association for the Advancement of Science. (b) The membrane is stretched with a three-dimensional strain induced by a microdiaphragm located at the bottom of the basolateral chamber.^59^

Micro-engineered systems with an integrated membrane in a microfluidic setting have been reported to model various *in-vivo* barriers, such as those of the lung alveoli, the brain, and the gut, to name a few.^57^ By implementing a flexible membrane in the microfluidic system, mechanical forces, such as those induced by the breathing motions, could be reproduced.^58,59^ Huh and colleagues reported an innovative microsystem made of a central chamber, itself divided by a thin, elastic, and porous membrane [Fig. 2(a)]. Lung epithelial cells and lung endothelial cells were cultured on both sides of the membrane to recreate the air-blood barrier at the air-liquid interface. The membrane can be stretched unidirectionally via the action by providing a vacuum in two adjacent chambers. The system was used to investigate the inflammatory response on lung epi- and endothelium upon exposure to nanoparticles and more recently to recreate drug toxicity-induced pulmonary edema.^60^ The system was further used to mimic the peristalsis movement of the gastro-intestinal barrier.^61^

A second lung-on-a-chip reproducing an array of three thin, stretchable, alveolar barriers was recently described by our group^58^ [Fig. 2(b)]. In contrast to the lung-on-a-chip reported by Huh and colleagues, the mechanical strain created is a three-dimensional strain, such as that taking place *in-vivo*. The breathing movements of the alveolar barrier are generated by applying a very small cyclic pressure in the medium located on the basolateral side of the alveolar membrane. The pressure variation is generated by the inflation and deflation of an actuation membrane (called micro-diaphragm) located in this small compartment and connected to an external electro-pneumatic generator. The alveolar membrane is made of an ultra-thin (3 *μ*m thin) polymeric membrane with an array of pores (either 3 or 8 *μ*m in diameter) on which cells are cultured. The results obtained with the device demonstrated that the strain influences the metabolic activity and the cytokine secretion of primary human pulmonary alveolar epithelial cells obtained from patients.

### C. Types of mechanical strain and their effects

The types of mechanical strain reproduced with the microfluidic platforms reported above cover a wide range: unidirectional, bi-directional (either in xy or in xz directions), and three-dimensional strain. It is either quantified in terms of linear (elongation) or surface deformation or in strain components: circumferential or radial. Most devices have been used to generate a cyclic mechanical strain of relatively small amplitude at a frequency comprised between 0.2 and 2 Hz. Some platforms produced a uniform and others a non-uniform strain (strain gradient). This large variety of strains makes cross-comparisons between experimental results from different laboratories very difficult. Several attempts to compare^62^ or to correlate^34^ the strains were made. Assuming an isotropic deformation, a uni-axial strain, *ε_LIN_*, (linear elongation) can be correlated with a bi-axial strain, *ε_SA_*, (surface expansion) using the following equation:^34^

$$\varepsilon_{SA}={\left(\varepsilon_{LIN}+1\right)}^{2}-1,$$ (1)

with $\varepsilon_{SA}=({SA}_{f}-{SA}_{0})/{SA}_{0}$ and $\varepsilon_{LIN}=(L_{f}-L_{0})/L_{0}$, *L_0_* and *L_f_* being the length before and after elongation and *SA_0_* and *SA_f_*, the surface area before and after expansion. Table II gives a numerical example of the correlation between these strains based on a 10% linear strain. In the cases of a two-dimensional deformation in x and z and of a three-dimensional deformation, the deflection variation, *Δz*, is negligible over the length scale of a cell (about 20 *μ*m) compared to the typical dimensions (from 500 *μ*m to several mm) of the substrate diameter. The surface expansion is thus similar to that taking place in a uni-axial and a bi-axial strain device, respectively.

**TABLE II.** Types of mechanical strain generated with microfluidic-based stretching platforms. Cross-comparison between types of strain is often difficult. As an example, a 10% linear strain is correlated for each strain type with its associated surface strain.

Type of mechanical strain (illustration of a stretched cell)	Type of mechanical strain	Linear strain (num. example)	Surface area strain
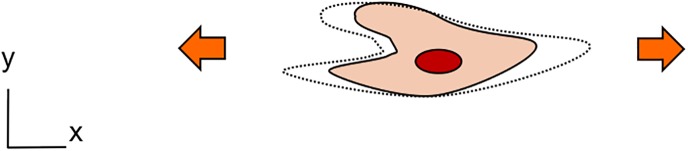	Uniaxial strain (x)	10%	10%
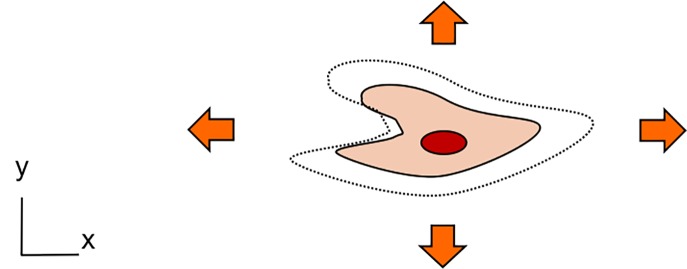	Bi-axial strain (xy)	10%	21%
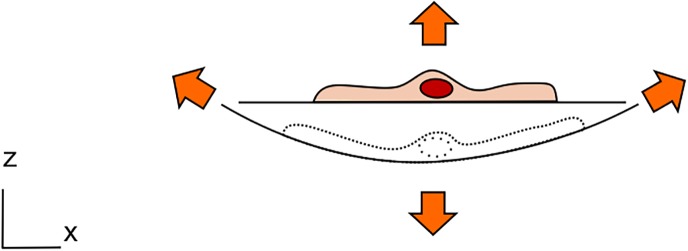	Bi-axial strain (xz)	10%	≈10%
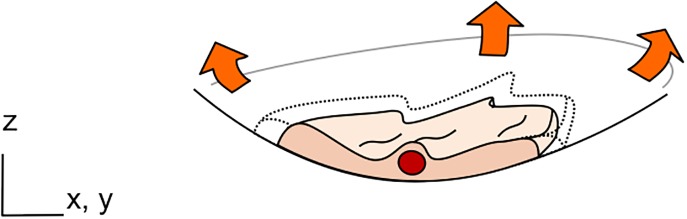	Tri-axial strain (xyz)	10%	≈21%

The various types of mechanical strain add to the complexity of the heterogeneous biological experimental procedures (types of cells, primary cells or cell lines, physiological medium, etc.). The first question to be answered is: Do all types of strain have the same effects on the cells? The second one is: How accurately does the *in-vivo* mechanical strain need to be reproduced *in-vitro* to trigger an *in-vivo*-like mechanoresponse? No straightforward and definite answers are yet provided to those questions.

Only a few studies cross-compared the effects of various types of mechanical strain. The cells respond to strain by morphological, biochemical, and genetic changes. The cells “feel” mechanical forces via their focal adhesion that transmit it to the cytoskeleton. As a result, the cytoskeleton remodels, which alters cellular morphology. The strain profile affects differently the cell's response. In airway smooth muscle cells, a uniaxial strain, but not a biaxial strain is a procontractile and proliferative stimulus.^63^ In vascular smooth muscle cells, a uniaxial, but not an equiaxial strain induces a transient increase in collagen I expression.^64^ Berry and colleagues reported about alterations in cellular alignment and of metabolic activity on adult and neonatal human dermal fibroblasts.^65^ The young fibroblasts showed greater cell proliferation and collagen production than adult dermal fibroblasts under unstrained conditions. More recently, Gould and colleagues investigated the effect of increasing anisotropy of biaxial strain on aortic valve interstitial fibroblasts.^66^ Cyclic biaxial strain in contrast to equiaxial strain induced both fibroblast proliferation and apoptosis and resulted in ECM reorganization. They suggested that cyclic equiaxial strain promotes a tendency towards a quiescent fibroblastic phenotype, and increasing strain anisotropy supports a tendency towards an active myofibroblastic differentiation. These results suggest that the type of mechanical strain importantly affects cells and should be carefully considered during the experimental design.

## IV. MECHANOTRANSDUCTION SIGNALING PATHWAYS INVOLVED IN MECHANICAL STRAIN

### A. Epithelia

**FIG. 3.**
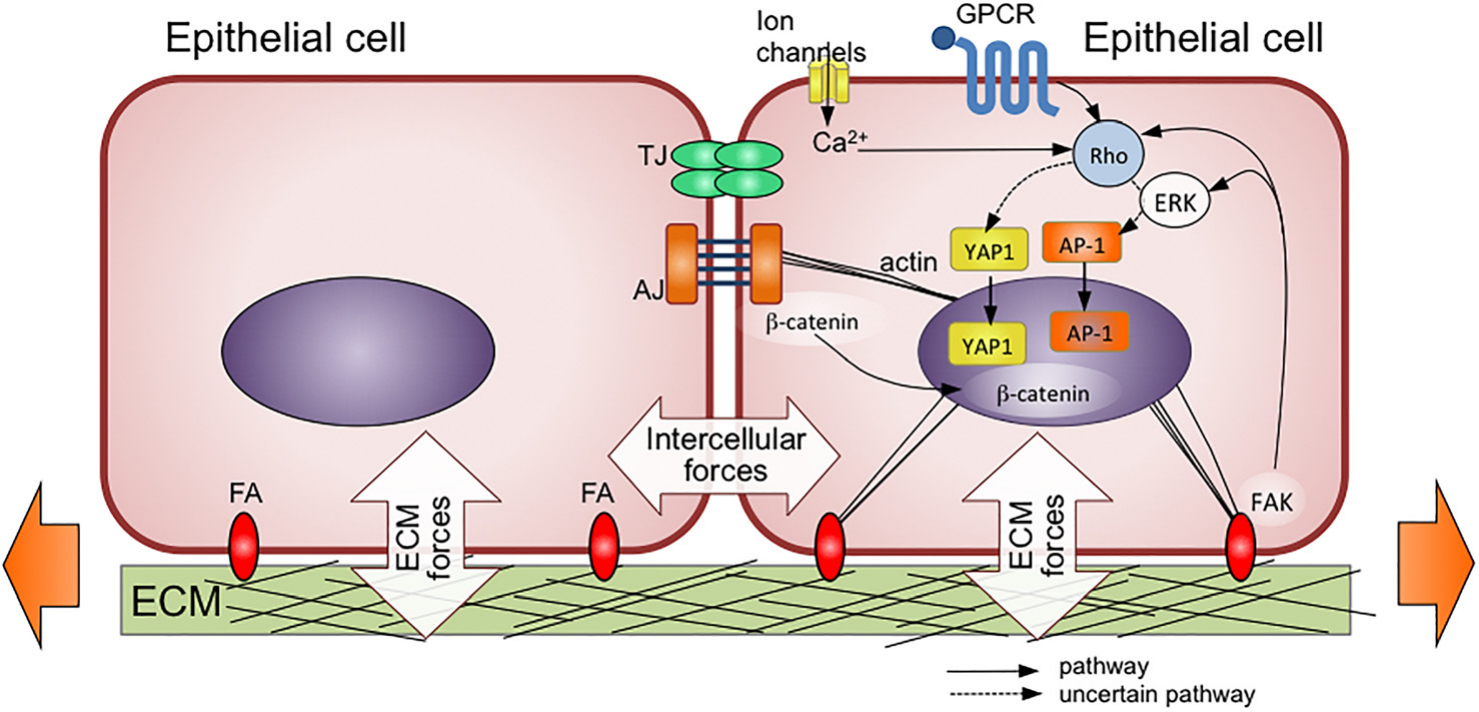
Forces transmitted via cell-cell and cell-extracellular matrix (ECM) attachments to epithelial cells during mechanical stretching. TJ = tight junction, AJ = adherens junction, FA = focal adhesion complex. Mechanical forces then activate several pathways that converge towards the activation of the transcription factors YAP1, AP-1, and β-catenin. Some of these pathways, such as GPCR-mediated activation of Rho signaling, are also activated by biochemical signals. Ultimately, mechanical and biochemical signals interact to lead to the observed physiological response. Dotted lines refer to pathways that are not entirely elucidated. Adapted with permission from Wang *et al.*, Cell Mol. Life Sci. **72**, 2091–2106 (2015). Copyright 2015 Springer Nature.

Several mechanosensitive pathways have been identified, which are summarized in Fig. 3. Some of the main inputs for mechanosignals include ion channels, G-protein coupled receptors, cell-ECM focal adhesions, and intercellular adherens junctions.^67^ Most of these pathways converge towards the cytoskeleton as a mediator of mechanical signals, although some bypass it altogether. Collectively, these inputs lead to the activation of three major transcription factors, namely, YAP, AP-1, and β-catenin.

YAP (Yes-associated protein) and its associated co-activator TAZ (transcriptional co-activator with PDZ-binding motif, also known as WWTR1) have received a great deal of attention due to their ability to regulate organ size and sense cell crowding.^68,69^ After tissue wounding, the spatial void allows cell spreading to occur, and epithelial cell spreading promotes nuclear translocation of YAP and its associated co-activator TAZ.^70^ In the particular case of the skin epidermis, YAP-mediated activation of transcription promotes the proliferation of keratinocytes as well as epidermal progenitors, while inhibiting terminal differentiation.^70,71^ Restoration of normal cell density and packing after reconstitution of the epithelial lining downregulate YAP signaling and enable return to homeostasis.

The AP-1 family of transcription factors is known to play a role in various skin diseases as well as during wound healing.^72^ AP-1 has been reported to be upregulated in stretched skin; however, the mechanism of activation is not yet understood. Another mechanosensitive pathway involves β-catenin, which is bound to the cytoplasmic domain of the intercellular adhesion molecule E-cadherin. β-catenin exhibits an armadillo repeat region that undergoes conformational changes upon mechanical stretching.^73^ Upon activation, β-catenin accumulates in the nucleus and causes transcription of target genes of the Wnt pathway, which promote cell proliferation and migration, both of which are inherently involved with the wound healing response.^74^

### B. Connective tissue

Epithelial barriers are generally supported by underlying connective tissue rich in ECM, blood vessels, and fibroblasts. In the case of skin, the dermis is profoundly affected by mechanical forces, especially after injury during wound repair. In the case of the lung, few fibroblasts are present under normal healthy conditions, but their prevalence increases dramatically during fibrosis.

**FIG. 4.**
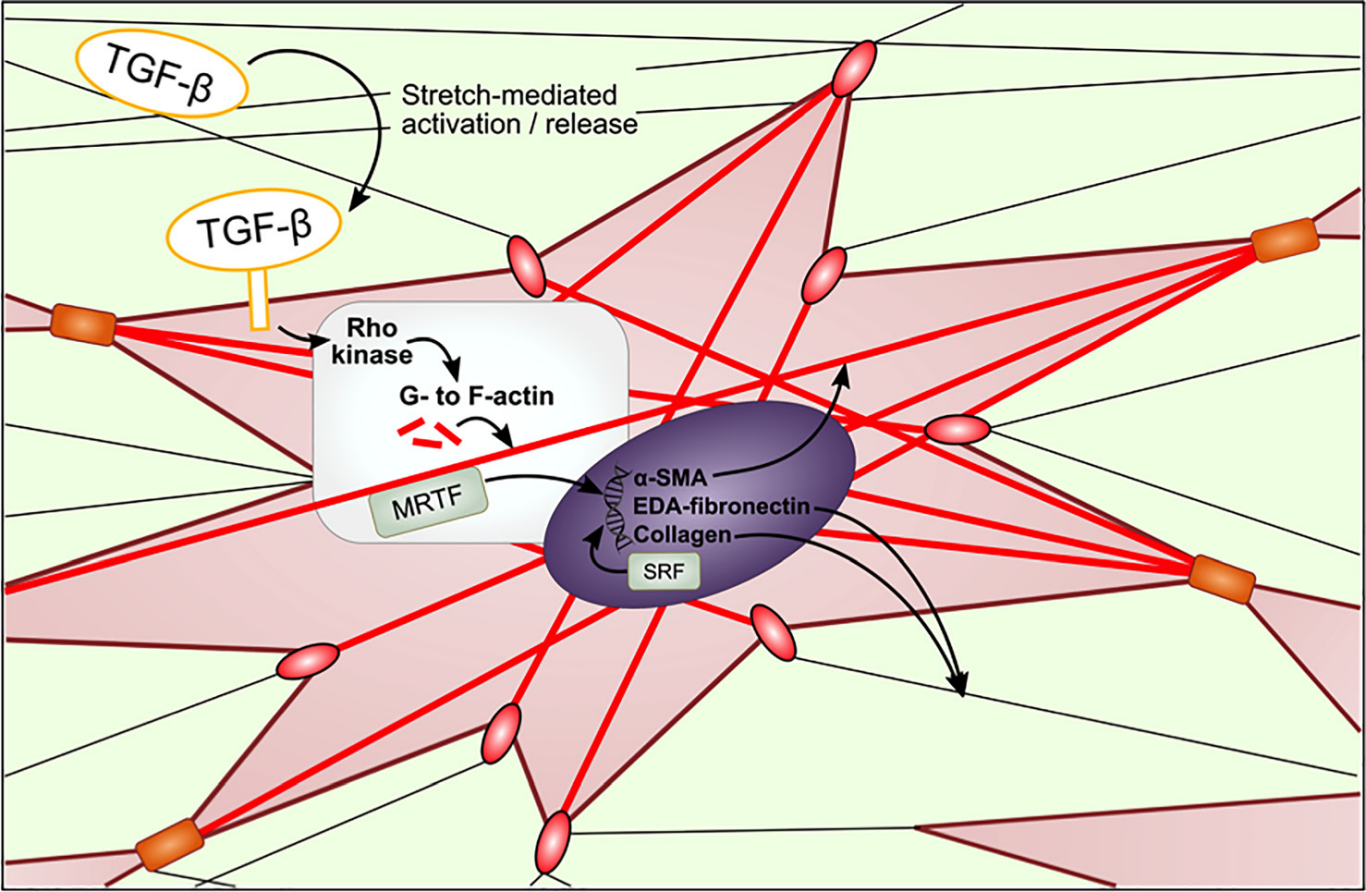
Mechanotransduction pathways in fibroblasts embedded in ECM. Fibroblasts interact with ECM through focal adhesion complexes (red ovals) and other fibroblasts via adherens junctions (orange rectangle). Red lines represent cytoskeletal stress fibers, which are initially made of F-actin, but incorporate alpha-smooth muscle actin (α-SMA) as fibroblasts differentiate into myofibroblasts. The pathways shown here are discussed in the text in more detail.

The Van de Water group summarized the mechano-stimulated pathways in fibroblasts, and their interaction with TGF-beta, the main pro-fibrogenic growth factor.^75^ Individual fibroblasts are part of a network of ECM comprising mainly of collagen but also fibronectin and other proteins. Transmembrane focal adhesions consisting of integrin clusters link the ECM network to the intracellular cytoskeleton. Fibroblasts also form intercellular adhesions with other fibroblasts via cadherins, from which also originate cytoskeleton fibers (Fig. 4).

Wong *et al.* distinguishes two types of mechanotransduction: extracellular and intracellular.^76^ In the former case, mechanical forces alter the way the ECM “presents” bioactive groups towards cells, by exposing hidden domains and/or physically altering their spatial density. Furthermore, a mechanism whereby surface-bound TGF-β can be released into solution by subjecting the ECM to stretch has been described.^77^ TGF-β signaling promotes the formation of F-actin from unpolymerized G-actin. In this intracellular process, the myocardin-related transcription factor (MRTF) is released from G-actin, which then enters the nucleus and joins with the constitutively expressed serum response factor (SRF), to trigger the expression of alpha-smooth muscle actin (α-SMA), EDA-fibronectin, and collagen.^75^ The appearance of α-SMA, which combines with the actin stress fibers, signals the differentiation of fibroblasts into myofibroblasts with enhanced contractile force. EDA-fibronectin, a splice variant of fibronectin specifically expressed after injury and in fibrotic disorders, enhances pro-fibrotic effects of TGF-β. Mechanical tension, via focal adhesions, also activates F-actin polymerization, and synergizes with TGF-β signaling intracellularly. Although not shown in the figure above, a recent report suggests that MRTF is a key player in the activation of TAZ in the YAP/TAZ pathway in fibroblasts.^78^ Activation of YAP/TAZ promotes the expression of TGF-β and the connective tissue growth factor (CTGF), which favor ECM deposition and crosslinking.^79^

Mechanical stress also activates YAP/TAZ, highlighting its role as a master regulator of mechanotransduction.^79^ NIH/3T3 cells exhibit increased YAP/TAZ activation when cultured in stiff ECM, but not soft ECM, which promotes pro-fibrotic pathways of ECM production and contraction.^80^ YAP/TAZ are highly expressed in fibrotic lesions but not normal healthy areas of the lung.^81^ An interplay between TGF-β and YAP/TAZ has also been implicated in pro-fibrotic pathways underlying diseases in lung^82^ as well as in other tissues.^83^ Fibrotic lesions, through activation of YAP/TAZ, may also promote epithelial to mesenchymal transition, as well as loss of contact inhibition, which are associated with carcinogenesis.^68^

## V. CONCLUSION

The advent of microfluidics and the renewed interest in mechanobiology have inspired researchers to develop new types of microfluidic devices able to reproduce *in-vitro* the *in vivo* mechanical strain. Results from the literature suggest that the strain type (1-D, 2-D, or 3-D), magnitude, and frequency can greatly influence the morphology and the metabolism of cells. Therefore, the strain parameters should be given as much attention as the choice of culture medium, hormone and growth factor supplements, or any other experimental parameters of the cell culture.

From a technological point of view, more advanced microfluidic-based platforms incorporating mechanical strain will very likely be developed in the years to come. They will combine strain, shear forces, stiffness, and additional features in a high throughput format. Furthermore, sensors can be integrated in the devices to monitor the mechanical strain,^84^ or extract biochemical information from the system,^85^ and actuators can electrically and/or mechanically stimulate cells.^86^

The ability to implement *in-vivo*-like strain in cell culture environments will enable a precise and thorough investigation of the mechanobiology of tissues exposed to strain. Besides understanding the effect of mechanical forces on cells, such studies will also provide new information on how mechanical and biochemical signals interact within signal transduction pathways to elicit cellular responses. This may open the way to a new discipline, mechanopharmacology, which will target specific pathways associated with mechanical strain.^87^
